# 

**Published:** 1894-01

**Authors:** 


TABLE OF CONTENTS ON LAST ADVERTISING PAGE.

VOL. IX. JANUARY, 1894. No. 7.

LIBRARY
[illegible]
S. G. O.

### TEXAS

### Medical Journal.

SUBSCRIPTION, $2.00 A YEAR, IN ADVANCE.
SINGLE COPIES, 25 CENTS.

PUBLISHED AT AUSTIN, TEXAS, BY

F. E. DANIEL, M. D., & S. E. HUDSON, M. D.,
EDITORS AND PROPRIETORS.

Entered at the Postoffice at Austin, Texas, as Second-class Mail Matter.

